# Supplementary material for: The genome sequence of the Silver Colonel, Odontomyia argentata (Fabricius, 1794) (Diptera: Stratiomyidae)
Source: Wellcome Open Res. 2026 Jun 27;11:398. [Version 1] doi: 10.12688/wellcomeopenres.26887.1 (PMC13560865; doi:10.12688/wellcomeopenres.26887.1)
Supplement: Peer review history

# Reviewer report: Reviewer response for version 1

**DOI:** 10.21956/wellcomeopenres.29606.r159827

**Published:** 2026-08-04

**Authors:** Himanshu Dubey

**Affiliations:** 1 Seribiotech Research Laboratory, Bangalore, Karnataka, India

The manuscript presents a high-quality chromosome-level genome assembly of *Odontomyia argentata* and provides a valuable genomic resource for future studies in comparative genomics, evolutionary biology, and insect genetics.

 The sequencing strategy, assembly pipeline, and quality assessment follow current best practices, and the assembly metrics demonstrate a robust and reliable reference genome.

 As a genome resource/data note, the scope of the study is appropriate, and the analyses presented adequately support the reported assembly and associated datasets.

 Overall, the manuscript represents a valuable contribution to the genomic resources available for Diptera and will serve as a useful reference for future research. I recommend the manuscript for acceptance.

Are sufficient details of methods and materials provided to allow replication by others?

Yes

Is the rationale for creating the dataset(s) clearly described?

Yes

Are the datasets clearly presented in a useable and accessible format?

Yes

Are the protocols appropriate and is the work technically sound?

Yes

Reviewer Expertise:

Genomics

I confirm that I have read this submission and believe that I have an appropriate level of expertise to confirm that it is of an acceptable scientific standard.

# Reviewer report: Reviewer response for version 1

**DOI:** 10.21956/wellcomeopenres.29606.r160567

**Published:** 2026-07-13

**Authors:** Darren Obbard

**Affiliations:** 1 The University of Edinburgh, Edinburgh, Scotland, UK

This data note reports the sequencing and assembly of the genome of Odontomyia argentata as part of the “Darwin Tree of Life” programme.

 In common with other data notes from this research effort, the reporting is standardised and quite brief. As such, I have very few comments to make.

 The approach is state-of-the-art, the raw data appear to be of a suitably high quality, and the assembly methods are appropriate.

 The public availability of raw data and genome assembly are appropriate. The resulting genome is likely to be of very high quality, and I have no doubt that it will be of great value to any researchers working on this fascinating group of flies, or on the comparative or evolutionary genomics of insects more generally.

 I have no substantive comments to make, just

 (1)    Paragraph 1 in the ‘Background’ – please avoid starting a sentence with an abbreviation “O.”

 (2)    ‘Background’ Paragraph 3, “… Nationally Scare based on its distribution and is also listed as a species ..” – I think there should be a comma before the ‘and is also listed’. Also, since I don’t know the technical definition of “nationally scarce” this sentence is hard to follow; is being of both ‘Least concern’ and ‘Nationally scarce’ as self-contradictory as it sounds?

 (3)    “It will aid research on …”, I think the value goes far beyond the species or even the family – for example, studies like this enable studies such as:  https://www.biorxiv.org/content/10.64898/2026.06.01.729285v1

Are sufficient details of methods and materials provided to allow replication by others?

Yes

Is the rationale for creating the dataset(s) clearly described?

Yes

Are the datasets clearly presented in a useable and accessible format?

Yes

Are the protocols appropriate and is the work technically sound?

Yes

Reviewer Expertise:

Evolutionary and comparative genomics of invertebrates and their pathogens

I confirm that I have read this submission and believe that I have an appropriate level of expertise to confirm that it is of an acceptable scientific standard.
